# Insect education and outreach: integrating traditional and non-lethal collection techniques

**DOI:** 10.1093/jisesa/ieag052

**Published:** 2026-06-18

**Authors:** Michelle G Au, Akriti Adhikari, Alexis Marie Alsdorf, Nathan C Arey, Haylie J Brown, Abigail L Cohen, Sabrina A Elgar, Eleanor Fausett, Judicaël Fomekong-Lontchi, Taylor Green, Dowen Mae Jocson, Kushal Naharki, Kyra Peterson, Kennedy White

**Affiliations:** Department of Plant and Environmental Protection Sciences, University of Hawaiʻi at Mānoa, Honolulu, HI, USA; Department of Agricultural Sciences and Engineering, Tennessee State University, Nashville, TN, USA; Department of Entomology and Plant Pathology, North Carolina State University, Raleigh, NC, USA; Department of Entomology and Plant Pathology, University of Tennessee, Jackson, TN, USA; School of Plant and Environmental Sciences, Virginia Tech, Blacksburg, VA, USA; Department of Entomology, Michigan State University, East Lansing, MI, USA; Department of Entomology, University of Georgia, Athens, GA, USA; Department of Ecology, Behavior and Evolution, University of California at San Diego, La Jolla, CA, USA; Department of Biology, Brigham Young University, Provo, UT, USA; School of Life Sciences, Oregon State University, Corvallis, OR, USA; Department of Entomology, Washington State University, Pullman, WA, USA; Department of Plant and Soil Sciences, West Virginia University, Morgantown, WV, USA; Entomology and Nematology Department, University of Florida, Gainesville, FL, USA; Department of Entomology, University of California Riverside, Riverside, CA, USA

**Keywords:** natural history, taxonomy, stewardship, ethics, science communication

## Abstract

Insect collections offer a unique and practical way to enrich students’ education at every level. Collecting insects and identifying specimens provides an active, hands-on activity that connects students to the natural world in a way that lectures alone cannot. With the advancement of technology, non-lethal approaches to traditional museum collections can still provide an experiential learning opportunity without the need for ethical considerations. This review emphasizes the significance of insect exposure in primary school, secondary school, undergraduate instruction, and public outreach settings, utilizing both traditional and contemporary methods. The goal at all levels is to share information about the diversity and uniqueness of insects while reducing the fear and stigma surrounding entomology. While traditional insect collections have justifiable applications, newly developed non-lethal methods are just as valid. Determining the most suitable methods for the target audience is crucial for the future of entomology. This article is part of the 2025 Collaborative Publication Program organized by the Entomological Society of America Student Affairs Committee to address emerging issues in Entomology.

## The Evolution of Insect Collecting: From Curiosity to Culture

The practice of collecting and preserving insects has deep historical roots, tracing humanity’s enduring fascination with the complexity of the natural world. Aristotle produced one of the earliest known systems of classification, grouping insects by traits such as wings and mouthparts, an approach that influenced later works, including Pliny’s *Historia Naturalis* (Beier 1973). During the Renaissance, insects found a place in cabinets of curiosity, prized both for their aesthetic beauty and as symbols of wealth and intellect (Osborn 1952). These collections merged art and science, reflecting early efforts to bring order to nature while celebrating its beauty and diversity (Beier 1973).

**Fig. 1.**
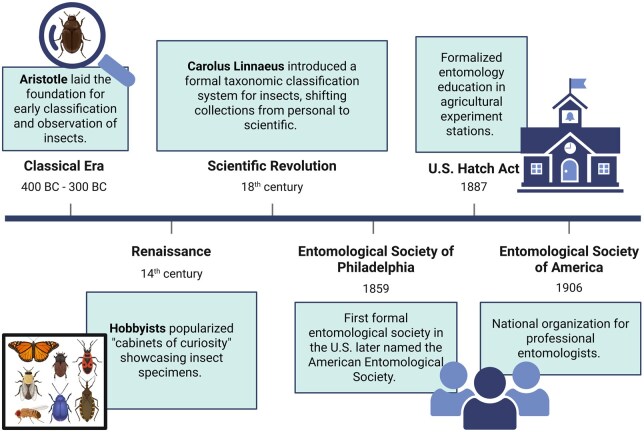
Timeline illustrating key milestones in the development of natural history, taxonomy, and insect collections from the Renaissance (ca. 1350–1600) through the Scientific Revolution (ca. 1543–1710) and the Enlightenment, including the contributions of Carolus Linnaeus (1707–1778). Early periods emphasize the foundations of classification and specimen study, while later entries highlight the growth of formal collections and their integration into academic and professional institutions, particularly in the United States. Adapted from Cresson (1911) and Leather (2015). Created in BioRender. Brown, H. (2026) https://BioRender.com/56fjk8g.

By the 18th century, systematic taxonomy transformed insect collecting from private amusement to scientific enterprise (Koerner 1999). Carolus Linnaeus introduced a universal system of binomial nomenclature (Winsor 1976), and Johan Christian Fabricius expanded upon it, emphasizing mouthpart morphology as a diagnostic feature (Leather 2015). These frameworks made it possible to classify and compare insects globally, laying the foundation for modern entomology. In the 19th century, the establishment of the Société Entomologique de France (1832) and the Royal Entomological Society of London (1833) institutionalized the discipline. On February 22, 1859, the Entomological Society of Philadelphia became the first entomology-focused society in the United States (Cresson 1911). As of 1887, the U.S. Hatch Act formalized entomology education in agricultural experiment stations (Leather 2015). As interest in the advancement of entomology grew, the Entomological Society of Philadelphia was recognized across the USA, with its name changed to the American Entomological Society in 1906 (Cresson 1911, Leather 2015). Important historical figures like Charles Darwin exemplified the value of this hands-on practice in scientific development through his beetle collections that underpinned his later theories. Figure 1 graphically depicts the transition from Aristotle to the formation of Entomological Societies as they stand today.

Insect collecting soon became a cornerstone of biological education. The breadth of collections across academic and museum institutions underscores their role in serving as tangible records of biodiversity and training tools for students to practice taxonomy, curation, and observation. Over time, however, the motivations behind collecting evolved from the pursuit of beauty and rarity to scientific documentation, and more recently, to ethical reflection. Contemporary educators increasingly reconsider the role of lethal collection in light of conservation priorities and changing attitudes toward animal welfare. Simultaneously, advances in imaging, digital databases, and artificial intelligence have made it possible to study insect anatomy and ecology without the need to kill or preserve specimens (González-López et al. 2021, Rodway and Schepman 2023).

Yet, as society grows more reliant on technology, physical engagement with nature has diminished. Traditional insect collection offers a sensory, embodied experience that digital tools cannot replicate. Handling, pinning, and curating insects cultivate patience, dexterity, and fine observation skills, qualities foundational to taxonomy and museum curation (Novack and Goldin-Meadow 2017, Sitar et al. 2023). Moreover, such experiences nurture empathy and curiosity toward living organisms, serving as a counterbalance to increasingly abstract modes of learning (Lindemann-Matthies et al. 2011).

As educational philosophy and conservation ethics continue to evolve, entomology must navigate the balance between traditional collecting and modern innovation. This review explores that evolution, examining whether insect collecting and specimen preservation should remain central in kindergarten through 12th grade (K-12) education, undergraduate instruction, and public outreach, or give way to more sustainable and technology-assisted approaches.

## Insect Collections in Primary and Secondary (K-12) Education

Active learning, defined by Handelsman et al. (2007) as students participating and moving in the classroom, is an approach structured to make students drivers in their own education. By fostering meaningful engagement with the material, active learning has been shown to increase student understanding, enhance retention, and build scientific skills (Handelsman et al. 2007, Grando et al. 2018, Tutal and Yazar 2023). In today’s technology-driven learning environments, K-12 students are more likely to have limited interactions with their natural environment and are consequently less invested in local species (Lindemann-Matthies et al. 2011). Across the globe, primary and secondary students have access to local insects that are both abundant and viable tools for teaching Science, Technology, Engineering, and Mathematics (STEM) education. Insect collections are a way to incorporate a fraction of these insect populations effectively.

Sitar et al. (2023) proposed a framework to introduce children in the third and fourth grades (ages 8 to 10) to entomology and conservation; the 4 guiding principles of this framework are knowledge acquisition, skills development, sustainable actions, and positive attitudes. This framework also incorporates an introduction to lethal insect collection with these principles in mind. Insect collections introduce children to classification, identification, conservation, appreciation, the importance of ethical practices, and skills used by many scientists. However, for these younger learners, pinned specimens can convey that insects are expendable rather than dynamic organisms that interact with their environments in fascinating ways. Research in early childhood education shows that students still forming an understanding of death may find assignments involving killing animals confusing or troubling (Kane 1979, Villiers and Monk 2005). More constructive avenues at this stage include observing live insects in terrariums or school gardens, using magnifiers or microscopes to explore behavior or morphology, and documenting encounters with photographs and drawings (Matthews 1997, Prokop and Fančovičová 2016, Quashie-Williams 2019, Miller et al. 2025). Carefully structured inquiry with live organisms can also demonstrate how science learning shifts when children’s questions guide activities, rather than when teachers simply show insect displays and impart knowledge (Haefner et al. 2006).

Learning with insects is not just constrained to early biology. Insects are small and accessible, which allows them to be used as a tool to connect different subjects within STEM disciplines. Through middle and high school (ages 11 to 18), insects are already used to great effect in lessons on arthropod-vectored diseases, food webs, and behavior, often through case studies, observation, or classroom rearing (Anderson and Stubbs 1996, Golick and Heng-Moss 2013). Beyond observation of pinned specimens, a wide range of engaging alternatives exists. Students can rear mantids to study predation, observe ant colonies to investigate communication, or use orthopterans to explore acoustic behavior, all of which provide authentic learning without requiring lethal collections. Rearing insects such as mealworms has been used to explore choice behavior, maze learning, and communication, while live snail activities have been shown to improve children’s knowledge and reduce disgust sensitivity compared to lecture-only lessons (Matthews 1997, Prokop and Fančovičová 2016). Programs involving silkworms or rhinoceros beetles have also enhanced children’s emotional health and awareness of insects (Miller et al. 2025). Citizen science platforms like iNaturalist further extend opportunities by allowing students to document species in their own communities while contributing to global biodiversity databases (Spiesman et al. 2021). Gamified activities where students role-play life cycles, match insect parts and identities, and handle organisms, further foster enthusiasm and long-term interest (Tribull 2019, Miller et al. 2025).

Programs are beginning to incorporate both traditional and experiential learning into their curricula. For example, biomimicry is an environmental approach to engineering that uses structures and behaviors that appear in nature to inspire designs for human use (Goldner et al. 2021). “Designing Bugs and Innovative Technology” (D-BAIT) is an example of insect collections being used to introduce biomimicry to high school students (Han et al. 2020). In D-BAIT, students are tasked to create a fishing lure that suspends itself in the water column at a particular depth by studying the morphology and behaviors of aquatic insects. To do so, students both observe live insects in the field as well as reference specimens from an insect collection in order to collect both the morphological and biological components required for this project. D-BAIT’s immersive approach not only deepens students’ understanding of insect biology and biomimicry but also fosters creativity and critical thinking by bridging scientific observation with real-world design challenges (Han et al. 2020).

## Insect Collections in Undergraduate Entomology Courses

Undergraduate degree programs provide students with opportunities to engage in interdisciplinary study, enhancing communication skills and intellectual versatility by exposing students to content beyond their primary fields of interest (Jacobs and Frickel 2009). Such education improves both cognitive and noncognitive skills and correlates positively with post-graduation earnings, particularly in the scientific disciplines (Han et al. 2023). A valuable but often underappreciated area of interdisciplinary integration for students in the life sciences is the creation of insect collections and learning proper curation techniques, which bridges ecology, taxonomy, conservation, and science communication. The development of a comprehensive student insect collection is a staple of undergraduate-level entomology coursework that has persisted for over 100 years (Osborn 1911, Kumar et al. 2022). Insect collection and curation techniques remain relevant in today’s entomological coursework as they equip students with foundational hard and soft skills that are transferable between life science disciplines and valued by employers (Han et al. 2023, University of California, Riverside 2023, Holwell et al. 2025). Students have the opportunity to learn both soft skills, such as time management, collaboration, critical thinking, and problem-solving, in addition to hard skills, including data collection and management, microscopy, and specimen identification, when developing an insect collection. By collecting specimens, students engage directly with biodiversity, develop keen observational acuity, and grow a deeper appreciation for ecosystem complexity—experiences that cannot be replicated through textbooks or digital media alone (Weeks 2015).

Insect collections also serve as invaluable scientific repositories for taxonomy, systematics, and conservation research, enabling students to contribute to species identification, environmental monitoring, and preservation of endangered taxa (Gasper and Gardner 2013, Wycoff et al. 2024). There are numerous examples in the literature that highlight novel scientific discoveries in insect distribution, species descriptions, plant–insect associations, and pest management that directly result from data gathered during insect collection exercises on college campuses (Wheeler 1989, Magnum and Brooks 1997, Staines and Staines 2021). A recent report from Fischer et al. (2021) highlights the detrimental impacts of reduced lepidopteran whole-specimen insect collecting from both amateur and professional entomologists and discusses the limitations of non-lethal image-based insect cataloguing. Collection of insects, followed by proper curation and preservation, allows researchers to acquire morphological, molecular, spatiotemporal, and physiological data that is not always available through photographed images (Fischer et al. 2021).

The University of Georgia’s study abroad program in Monteverde, Costa Rica (https://globalengagement.uga.edu/study-abroad/), is a case study showing the benefits of an immersive student experience focused on insect collection, curation, and identification (Robinette and Noblet 2009). The curated specimens are donated to local schools, fostering a reciprocal exchange that benefits both students and communities while sharpening students’ bilingual scientific communication (Robinette and Noblet 2009). This model demonstrates how insect collection and curation cultivate hands-on expertise and cultural and environmental awareness alike. Integrating insect curation techniques, such as direct pinning, double mounting, slide mounting, and ethanol preservation, into college curricula offers foundational technical skills essential for biology and entomology students as they work towards careers in these fields (Kumar et al. 2022).

However, despite gaining skills in morphology, taxonomy, and field collection techniques, evidence suggests that courses centered around the assignment of each student to create an individual insect collection are poorly aligned with modern pedagogical frameworks. These types of activities largely assess the lowest levels of Bloom’s Taxonomy, which categorizes cognitive skills based on complexity. Creating an insect collection emphasizes recall and recognition rather than higher-order skills such as analysis, evaluation, and hypothesis development (Crowe et al. 2008, Orr et al. 2022). Insect curation also consumes large amounts of laboratory time, requires specialized materials which can be challenging to source, and assumes fine-motor skills that may disadvantage some students. Because each student is tasked with assembling a similar collection, redundancy is common, and the exercise rewards the number of specimens obtained rather than the quality of learning. This individualistic structure can foster competition as students race to acquire the same local species, a dynamic that can create inequities, erode collaboration in the classroom, or encourage academic dishonesty (Lucky et al. 2019). Alternatively, insect collection courses that promote collaboration, whether through group assignments, specimen sharing, or communal collecting events, can enhance participation and foster a sense of camaraderie amongst students. It is crucial to determine the optimal course structure and collection guidelines to maximize student engagement and retention of knowledge.

More intentional undergraduate course designs offer stronger outcomes. A curriculum framework known as backwards design encourages instructors to define specific learning goals, such as data literacy, ecological reasoning, and hypothesis generation, then build activities that target those outcomes (Bopardikar et al. 2022). The application of Course-based Undergraduate Research Experiences (CUREs) is particularly promising in achieving these learning goals. They embed students in collaborative projects that generate new data while cultivating higher-order competencies. Research shows that CUREs increase retention in STEM, strengthen scientific identity, and improves inclusivity for students from historically excluded groups (Cooper et al. 2017, Flowers 2020, Bradshaw et al. 2023, Niccum 2025). Goodwin et al. (2021) also reflected upon the failures experienced in CUREs by students as fostering authenticity and resilience, contrasting with the rote accumulation of pinned specimens.

Examples of entomology-focused CUREs illustrate these possibilities. Keas et al. (2024) developed a moth ecology project in which students posed research questions, built traps, and analyzed diversity data. Although their design still required killing moths, the framework demonstrates how authentic ecological inquiry can be integrated into introductory classrooms and could be adapted using non-lethal approaches. Other models involve students in documenting pollinators and plants through community science (Mech et al. 2022, Doll and Summers 2024, Doll et al. 2024); catch-and-release field projects and non-lethal traps provide an authentic sampling experience (Diekötter et al. 2006); and automated camera or sensor-based monitoring systems record insect visitation without capture (Zou et al. 2017, Johnson et al. 2020). Even preserved specimens, if necessary, can be sourced ethically, as car-killed insects have been validated as reliable indicators of abundance and diversity (Møller 2019). Digitized museum records allow undergraduates to investigate long-term trends in insect distributions and seasonality (Davis et al. 2023, Weller 2023), while interactive identification tools and 3D models provide training in morphology and taxonomy without destructive sampling and improve accessibility to viewing specimens without the need of a microscope (Seal et al. 2023, Callohuari et al. 2025). These resources also lend themselves to creative assignments that mirror authentic scientific work. For example, building an interactive Lucid Key for local insects could be assigned in a lab course. Students must learn diagnostic characteristics, test the usability of their keys with peers, and reflect on how scientists communicate biodiversity knowledge. Such methods redirect energy from competition toward collaboration and equip students with transferable skills in analysis, communication, and ecological reasoning.

However, many of these innovative examples still stem from having a detailed and comprehensive museum collection. Skills like pinning are vital in specimen preparation for research and museum curation, requiring mastery of technique, taxonomy, and meticulous attention to detail that are only attainable by traditional methods (University of California, Riverside 2023, Oregon State University Extension Service 2026). Manual tasks such as pinning employ kinesthetic learning strategies that greatly enhance cognitive development and long-term retention (Novack and Goldin-Meadow 2017). In an age dominated by artificial intelligence (AI), skills demanding tactile feedback, precise motor control, and human judgment are indispensable, as AI cannot replicate the nuanced decision-making involved in specimen handling (González-López et al. 2021, Rodway and Schepman 2023). Hands-on activities inaccessible to AI improve memory retention, foster creative problem-solving, and build professional confidence—essential for scientific expertise both in the lab and field. Outreach research further shows that repeated, authentic insect engagement enhances student interest, conservation awareness, and academic recruitment into entomology (Pitt and Shockley 2014, Sieg and Dreesmann 2021, Wycoff et al. 2024).

## Insect Collection for Outreach

Universities, museums, and government agencies have long recognized the power of outreach to connect people with insects. Since the first reported insect-themed event, the Insect Fear Film Festival launched at the University of Illinois in 1984, insect-themed public events have grown into a cornerstone of entomological engagement across the United States (Tribull 2019). These festivals and fairs use costumes, crafts, insect-inspired games, microscope stations, and opportunities to handle live organisms to transform community attitudes towards arthropods (Frazier 2002, Hamm and Rayor 2007, Pitt and Shockley 2014, Wycoff et al. 2024, Miller et al. 2025). Evidence suggests that such live interactions are particularly effective. For example, Virginia Tech University Entomology Department’s outreach event, Hokie BugFest, introduced Hokie BugFest on the Go, bringing Madagascar hissing cockroaches, mantids, and beetles into Virginia classrooms and measurably reduced the percentage of children who reported disliking insects (Miller et al. 2025). Similar results have been reported at other outreach events where direct contact with living insects increased positive perceptions and sparked conversations about their ecological importance (Pitt and Shockley 2014, Lucky et al. 2025).

By contrast, pinned collections displayed at outreach events often remain behind glass, offering little opportunity for interaction. While many individuals find the diversity and colors of a display box awe-inspiring, live insects like a beetle feigning death or hearing the hiss of a cockroach can leave a similar impact (Herbison 2025). Live engagement not only improves public attitudes but also highlights the ecological services insects provide, from pollination to nutrient cycling, strengthening stewardship values that extend beyond the outreach event.

This emphasis on stewardship is especially critical given mounting evidence of global insect declines. Populations are under pressure from habitat loss, climate change, invasive species, and a host of anthropogenic stressors (Jactel et al. 2020, Van Der Sluijs 2020, Dar et al. 2021, Wagner et al. 2021, Gebremariam 2024). Although only a handful of modern extinctions have been formally documented, thousands more are estimated to have occurred, particularly among specialists tied to narrow habitats or host plants (Dunn 2005). These trends represent not only an ecological crisis but also an educational responsibility. Continuing to encourage students to kill insects for learning could risk inadvertently harming vulnerable species and send conflicting messages about conservation efforts.

Although there is a possibility for students to collect endangered or vulnerable species, insect collections itself are not known to be detrimental to insect populations. The primary drivers of insect decline are changes to habitats and pollution (Raghavendra et al. 2022). These 2 factors are more likely to go unaddressed if humans are not conscientious and appreciative of insects. Negative associations with arthropods are especially true for those with arachnophobia and entomophobia. Fukano and Soga (2023) studied the increase of entomophobia in developed countries and how it may be contributing to the decline of insects from human activities. Fortunately, exposure reduces fear and individuals are likely to care more about insect conservation when they have interacted with a variety of insects (Grando et al. 2018, Shariari-Namadi et al. 2018, Wycoff et al. 2024). Insect collections encourage children to spend time outdoors, where they can become curious about their local ecosystems and familiarize themselves with animals that might otherwise become distasteful, all while gaining disciplinary skills and knowledge (Lindemann-Matthies et al. 2011). Integrating insect collection and curation practices into science education not only equips students with essential observational and technical skills but also fosters scientific curiosity and a deeper commitment to biodiversity as adults. These skills are foundational to entomological inquiry and environmental stewardship.

Across age groups and settings, the way insects are introduced matters. Entomology education cultivates positive attitudes and scientific literacy across diverse learners. Outreach programs employing insects to teach environmental concepts report increased student interest, intrinsic motivation, and teacher confidence regardless of delivery mode, highlighting the importance of live specimens in instruction (Weeks 2015). Hands-on interaction and thoughtful preparation successfully dispel negative biases about invertebrates, encouraging curiosity over fear (Weeks 2015). Diverse outreach modalities such as scientist-led classes, teacher training workshops, and online curricula offer complementary approaches that enrich science education with insect-centered activities. Teacher training enhances self-efficacy, which supports sustained entomology lessons within school curricula (Weeks 2015). Live outreach programs transform fear and misunderstanding into fascination and respect. At every level, education is not only about teaching entomology but also about shaping values toward stewardship. In the face of global insect decline and climate change, choosing approaches that cultivate respect for living organisms and emphasizing their ecological importance may be one of the most powerful contributions entomologists can make to both science and society.

## Ethical Considerations

Research on insect sentience and welfare has begun to close the divide between how vertebrates and invertebrates are treated in education and research (Fischer and Larson 2019, Lambert et al. 2021, Barrett et al. 2025, De Souza Valente 2025). However, insect euthanasia, curation, and preservation, conducted with ethical awareness and best practices, yield educational and scientific benefits. Ethical frameworks such as the 3Rs (Reduction, Refinement, Replacement) aim to minimize harm and increase respect for insect welfare while maximizing learning and research integrity (Godding 2025). Trietsch and Deans (2018) proposed “The Insect Collectors’ Code” as an ethical framework for individuals to recite and acknowledge before killing insects for general collecting or scientific sampling. The Insect Collectors’ Code introduces guidelines for the humane treatment of insects, minimization of ecological impacts, proper documentation, and validation of data. The growing recognition of insect sentience has also fostered the importance of humane insect collection practices that reflect evolving scientific understanding and promote respect for biodiversity (Gibson et al. 2015, Lambert et al. 2021). Ethical insect collection and curation guidelines advocate purposeful collecting, adherence to conservation principles, and the use of humane euthanasia methods, all effectively taught in college curricula to prepare conscientious scientists and biodiversity stewards (Barrett et al. 2025). Discussing ethically centered ideological frameworks prior to students initiating an insect collection also supports ethical reasoning development; students deepen their connection to nature, scientific literacy, and conservation awareness through hands-on collecting paired with stewardship discussions (Trietsch and Deans 2018, Fore and Barrett 2024). Conservation-focused education promotes population sustainability, cultivating scientists who balance research and preservation (Leather 2015, Ardoin et al. 2020). By combining interdisciplinary hands-on engagement with ethical mindfulness, insect collection and curation uniquely prepares students for meaningful careers and empowers biodiversity advocacy in an increasingly AI-driven era where direct natural interaction remains irreplaceable (Montero-Castaño et al. 2022, Barrett et al. 2025).

With technological advances, however, entomological research is shifting towards non-lethal methods such as mark-release-recapture, camera trapping, and non-lethal DNA sampling that bypass the need for ethical mindfulness while still supporting conservation education (Dunn 2005, Mola et al. 2021, Kirse et al. 2022). As Lövei et al. (2023) argue, entomology may be undergoing a methodological revolution similar to ornithology’s move away from the shotgun. Education should not lag behind this trajectory but instead lead by modeling non-lethal, inquiry-driven, and conservation-minded practices. Insect educational games, for example, can also be used as effective didactic tools in entomology classrooms. They have been shown to expose students to entomological topics and increase their knowledge, especially for noncharismatic insects (Cosme et al. 2020). Additionally, live interactions with specimens have been shown to improve engagement, learning outcomes, and attitudes towards often overlooked, non-charismatic organisms (Ballouard et al. 2012, Prokop and Fančovičová 2016). This positive shift in perception and better understanding of ecological importance can foster a stronger sense of stewardship and ethical responsibility. By incorporating non-lethal practices and interactive experiences, educators can nurture both scientific curiosity and ethical awareness in students. In doing so, the classroom becomes a space where conservation values are not just taught, but embodied.

## Conclusion

Insect collection, euthanasia, and curation have been long-standing practices for learning about the most speciose taxonomic group in the animal kingdom at every academic level. In recent years, these traditional practices have come under question by many individuals due to the rise in popularity of non-lethal insect collection and identification methods made possible by technological advances. This paradigm shift in the modern field of entomology has sparked debates amongst citizen and professional scientists about the ethical ramifications surrounding the collection and killing of insects for educational purposes in primary school, secondary school, college, and public outreach environments. Ethical frameworks for insect collectors have been formulated and adopted by many, but controversy surrounding these practices remains relevant and justified. Practitioners of the life sciences and educators must continue to explore and discuss the underlying ethical quandaries surrounding the collection and killing of insects. These traditional practices still have legitimate applications in the field of entomology and the world of insect education. However, newly developed non-lethal collection and identification methods are equally valid and can be beneficial additions or replacements to traditional practices in entomology lesson plans.

Determining the most suitable time and place to utilize these methods to serve the target audience is crucial for the future of entomology. The effective implementation of insect collections requires thoughtful consideration and careful planning. Educators can emphasize ethical considerations to their audience, such as the tenets of the Insect Collectors’ Code and the 3Rs (Reduction, Refinement, Replacement), before the start of insect collecting to ensure participants are aware of practices that promote insect welfare. If participants object to traditional insect collection techniques, alternative methods can be implemented, including the collection of already dead insects commonly found on cars, windowsills, and along pathways, or the killing of individual insects rather than mass sweep or trap samples. Alternatives that may offer more comprehensive learning opportunities compared to a traditional insect collection include observation and interaction with live specimens or insect rearing. The deliberate implementation of entomological education that emphasizes the humane and intentional understanding of insects through collections, rearing, experiments, and new technologies will provide a more robust learning opportunity and facilitate stewardship towards insects. The goal for both traditional and contemporary insect collection practices is to increase public awareness and reduce the negative stigma of these important and diverse organisms.
